# Primary Renal Glomus Tumor of Uncertain Malignant Potential: A Rare Case Report and Literature Review

**DOI:** 10.15586/jkc.v13i3.447

**Published:** 2026-09-23

**Authors:** Tongzhen Yan, Xuehao Zhou, Liqiang Guo

**Affiliations:** 1 Department of Urology, Qilu Hospital of Shandong University, Jinan, Shandong Province, China;; 2 Department of Urology, Shanghai General Hospital, Shanghai Jiao Tong University School of Medicine, Shanghai, China

**Keywords:** Kidney, Glomus tumor, Partial nephrectomy, Uncertain malignant potential

## Abstract

Renal glomus tumor (GT) is a rare neoplasm originating from glomus cells within the kidney. The subtype designated as uncertain malignant potential (UMP) is particularly rare, with limited literature available. Its clinical presentation and imaging features closely resemble those of renal cell carcinoma (RCC), making accurate preoperative diagnosis difficult. This case report aims to describe the clinical, radiological, and pathological features of a renal GT of UMP and to summarize previously reported cases to improve recognition of this rare entity. We report a case involving a 36-year-old male who was incidentally found to have a cystic-solid mass (5.8 × 5.1 × 4.2 cm) in the lower pole of the right kidney during evaluation for tinnitus. Contrast-enhanced CT suggested a diagnosis of RCC, prompting laparoscopic partial nephrectomy. Postoperative pathology revealed focal mild cellular atypia and a Ki-67 proliferation index of approximately 10%. Immunohistochemistry showed positivity for SMA and Collagen IV, focal positivity for CD34, and negativity for CD10, HMB45, Melan-A, Desmin, CK, and CK7. Based on histomorphology and immunoprofile, a diagnosis of primary renal GT with UMP was established. The patient recovered well postoperatively without further treatment, and no local recurrence or distant metastasis was observed during an 8-month follow-up. We reviewed the existing literature to summarize the clinical characteristics and diagnostic considerations of UMP-type renal GT, aiming to enhance recognition of this rare entity.

## Introduction

Glomus tumors (GTs) are rare mesenchymal neoplasms originating from glomus cells—modified smooth muscle cells surrounding arteriovenous anastomoses. They typically occur in the dermis or subcutaneous tissue, especially beneath the nail bed of extremities, while visceral involvement is exceedingly rare (1). Renal GTs are sparsely reported in the literature, and their clinical manifestations are nonspecific. Radiologically, they often resemble renal cell carcinoma (RCC), leading to frequent preoperative misdiagnosis.

Although most GTs are benign, recent studies have identified cases with cellular atypia, increased mitotic activity, or elevated proliferative indices, suggesting potential malignant behavior (2). In 2001, Folpe et al. proposed a classification system to identify atypical and malignant GTs (3), which was later adopted by the World Health Organization (WHO) in its 2013 classification of soft tissue and bone tumors. GTs are now categorized into benign, uncertain malignant potential (UMP), and malignant types (4,5). Renal GTs classified as UMP are extremely rare. Fewer than 10 cases have been reported in the literature. Their clinical behavior and management strategies remain poorly defined. This study presents a case initially misdiagnosed as RCC based on imaging, later confirmed pathologically as a UMP-type renal GT. We also review relevant literature to highlight its clinical features, diagnostic criteria, and follow-up recommendations, aiming to improve awareness and recognition of this rare tumor.

## Case Report

A 36-year-old male presented to the otolaryngology department with tinnitus. During systemic evaluation, contrast- enhanced abdominal CT incidentally revealed a cystic-solid mass in the lower pole of the right kidney, measuring approximately 5.8 × 5.1 × 4.2 cm. The lesion had well- defined margins and protruded beyond the renal contour. Mild patchy enhancement was observed at the lesion’s periphery during the corticomedullary phase, with decreased enhancement during the venous and excretory phases. RCC was considered the most likely diagnosis. (Figure 1A–C). The patient had no urinary symptoms such as frequency, urgency, dysuria, hematuria, or flank pain. He had no prior surgeries or family history of hereditary diseases.

**Figure 1:**
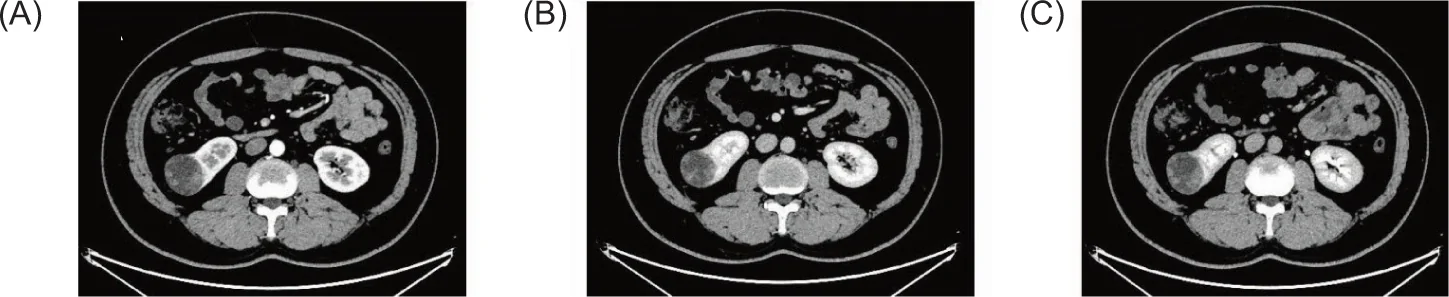
Axial contrast-enhanced CT images of the abdomen in three phases. (A) Arterial phase: Patchy enhancement is observed at the margin of the right renal lesion, and the internal portion of the lesion shows hypodense. (B) Venous phase: The degree of enhancement is reduced, with no evidence of invasion into the renal medulla or the collecting system. (C) Excretory phase: No contrast excretion is observed within the lesion. The boundary between the lesion and the renal pelvis is well defined.

Given the imaging findings suggestive of RCC, the patient underwent laparoscopic partial nephrectomy. Intraoperatively, a cystic mass was identified at the lower pole of the right kidney, with intact capsule and clear boundaries. The lesion protruded outward, and upon incision, the cyst was filled with fish-flesh-like tissue (Figure 2A,B).

**Figure 2:**
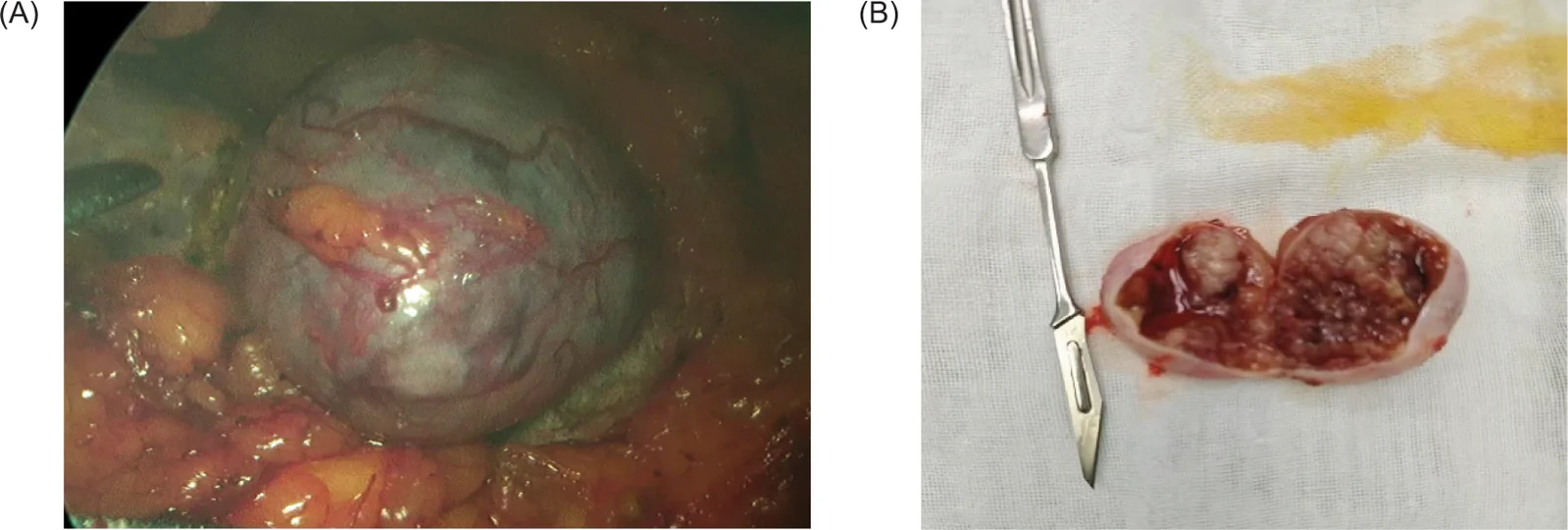
Gross appearance of the renal tumor. (A) Intraoperative view showing a cystic mass located at the lower pole of the right kidney. The capsule is intact, with well-defined margins and an outward protrusion. (B) Cut surface of the resected specimen reveals a cystic tumor measuring approximately 4 × 3.7 × 3.5 cm, with the cavity filled with fish-flesh-like tissue.

Histological examination (H&E staining) revealed nests of tumor cells with abundant cytoplasm and round to oval nuclei. The stroma contained vascular septa, and focal areas showed mild cellular atypia. No significant necrosis or active mitotic figures were observed (Figure 3). Immunohistochemical staining showed positivity for SMA and Collagen IV, focal positivity for CD34, and negativity for CD10, HMB45, Melan-A, Desmin, CK, and CK7. The Ki-67 proliferation index was approximately 10%. Based on the histological and immunohistochemical findings, the final diagnosis was a renal GT with features suggestive of UMP. Only short-term follow-up was initially available; however, the patient has now been followed for 8 months without evidence of recurrence or metastasis.

**Figure 3:**
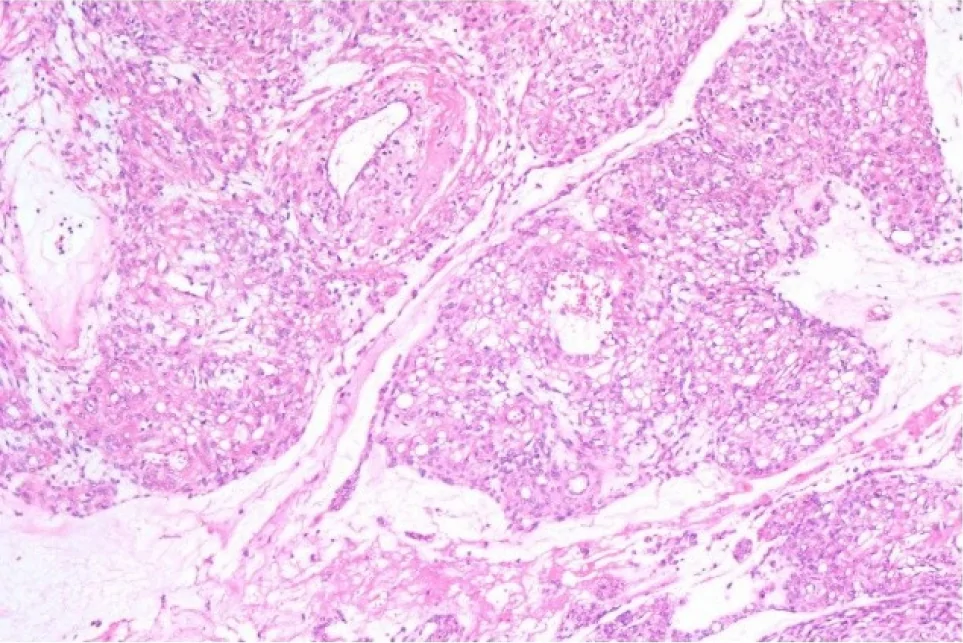
Hematoxylin and eosin-stained section of the tumor. The tumor cells are arranged in solid sheets and nests. They are relatively uniform in morphology, predominantly round or oval in shape, with eosinophilic cytoplasm and centrally located nuclei. Vascular structures are present within the tumor. Focal cytologic atypia is observed in some tumor cells.

## Discussion

GTs are exceedingly rare mesenchymal neoplasms derived from perivascular cells. They typically occur in the dermis or subcutaneous tissue and are rarely found in visceral organs, with primary renal GTs being particularly uncommon. Clinical manifestations of renal GTs are nonspecific and may include hematuria, flank pain, or incidental detection during imaging (6,7). Most cases present as well-defined, heterogeneously enhancing solid or cystic-solid masses on imaging, often mimicking RCC (8,9). In this case, the imaging findings were typical of RCC, leading to preoperative misdiagnosis.

**Table 1:** Summary of reported cases of renal glomus tumors of uncertain malignant potential.

Reference/Year	Age	Sex	Clinical presentation	Location	Size (cm)	Ki67	Atypia/Mitosis	Classification	Treatment
Gill et al (2010) (11)	46	M	Microscopic hematuria	Right kidney	7.0 × 6.5 × 6.5 cm	10%	No significant atypia/no atypical mitosis	UMP	Radical nephrectomy
Kapogiannis et al (2021)	67	M	Microscopic hematuria	Right kidney	2.7 × 2.1× 2.6 cm	1%	Mild pleomorphism/mitotic activity not confirmed	UMP	Robotic partial nephrectomy
Allen et al (2024) (2)	69	F	-	Right kidney	7.8 x 7.0 x 5.6 cm	-	Small cells with marked atypia/increased mitotic activity	UMP	Radical nephrectomy
Li et al (2018) (10)	33	F	-	Left kidney	9.5*6.0cm	-	Nuclear variation	UMP	Radical nephrectomy
Zhao et al (2018) (13)	8	F	-	Right kidney	5 cm	10%	Moderate nuclear atypia/no abnormal mitosis	UMP	Partial nephrectomy
Present case (2025)	36	M	-	Right kidney	4 × 3.7 × 3.5 cm	10%	Focal mild cellular atypia	UMP	Partial nephrectomy

Histologically, benign renal GTs are composed of densely arranged, round cells with abundant cytoplasm and rich capillary networks. The cells are morphologically uniform, with rare mitotic figures and low proliferative indices (3,10). However, recent reports have described renal GTs with cellular atypia, increased mitotic activity, and elevated Ki-67 indices, suggesting biological activity or malignant potential (11). In this case, the presence of mild nuclear atypia and a Ki-67 proliferation index of 10%, in the absence of definitive malignant histological features, supports classifying the tumor as a glomus tumor of uncertain malignant potential (UMP).

Immunohistochemistry plays a crucial role in differentiating renal GTs. GTs typically express smooth muscle markers such as smooth muscle actin (SMA) and type IV collagen, indicating their origin from perivascular smooth muscle-like cells (12,13). They are generally negative for epithelial markers, including CK, CD10, and CK7, which helps exclude RCC, as RCC often expresses CK, CD10, and PAX8 (14,15). In addition, GTs lack melanocytic markers such as HMB45 and Melan-A, which are strongly expressed in perivascular epithelioid cell tumors (PEComas) (16).

Although most GTs are benign, some cases exhibit aggressive biological behavior. To assess malignant potential, Folpe et al. proposed criteria in 2001, including deep location, size >2 cm, atypical mitotic figures, or ≥5 mitoses per 50 high-power fields (HPFs). Tumors lacking overt malignant features but exhibiting high mitotic activity or large size in deep locations are classified as UMP (3). This classification was adopted by WHO in its 2013 and 2020 editions of the soft tissue and bone tumor classification (4,5).

Globally, fewer than 10 cases of UMP-type renal GTs have been reported, mostly as case reports. A brief literature search was performed in PubMed using the terms “renal glomus tumor” and “glomus tumor of uncertain malignant potential.” Most reported tumors were incidentally detected and showed imaging features mimicking RCC. Pathologically, previously reported UMP-type tumors commonly demonstrated mild to moderate atypia with variable Ki-67 proliferation indices, consistent with the findings in our patient. The physical examination in our patient was unremarkable, which is consistent with most previously reported renal GTs that lack specific clinical signs. The primary differential diagnosis is RCC, as both entities typically present as incidentally detected renal masses with overlapping imaging features. In this case, the well-circumscribed enhancing mass on CT closely resembled RCC, making pre-operative distinction difficult. Therefore, definitive diagnosis relies on histopathology and immunohistochemistry. These cases span a wide age range and affect both sexes. The present case involves a relatively young patient (36 years old) with a moderately sized tumor and an elevated Ki-67 index, aligning with previously reported UMP-type cases. A summary of reported cases is provided below.

In summary, the present case aligns with previously reported renal GTs in terms of clinical presentation, imaging, and histopathology. Importantly, this case highlights the extreme rarity of UMP-type renal GTs, with fewer than 10 cases reported worldwide. The patient’s young age and the successful use of nephron-sparing surgery further underscore the clinical significance of this report, as kidney preservation is particularly valuable for long-term renal function in younger individuals. Although the tumor exhibited mild atypia and an elevated Ki-67 index, no recurrence or metastasis has been observed during 8 months of postoperative surveillance. Continued long-term follow-up remains essential to better understand the biological behavior of this rare entity.

## Conclusion

This tumor was located in the lower pole of the right kidney and was completely excised during surgery. The patient recovered well postoperatively, and 8-month follow-up revealed no recurrence or metastasis, demonstrating the effectiveness of partial nephrectomy in treating GTs. Histological findings indicated a degree of malignant potential, underscoring the importance of close postoperative surveillance.

## Mandatory Disclosure on Use of Artificial Intelligence

The authors used artificial intelligence tools (ChatGPT/ Copilot) only for language polishing and grammar improvement. No AI tools were used for data analysis, interpretation, or generation of scientific content. All final decisions and revisions were made by the authors.
